# E670G PCSK9 polymorphism in HeFH & CAD with diabetes: is the bridge to personalized therapy within reach?

**DOI:** 10.3389/fcdhc.2023.1277288

**Published:** 2023-11-01

**Authors:** Rano Alieva, Aleksandr Shek, Alisher Abdullaev, Khurshid Fozilov, Shovkat Khoshimov, Guzal Abdullaeva, Dariya Zakirova, Rano Kurbanova, Lilia Kan, Andrey Kim

**Affiliations:** ^1^ CAD & Atherosclerosis Department, Republican Specialized Center of Cardiology, Tashkent, Uzbekistan; ^2^ Center for Advanced Technologies, Ministry of Innovative Development of Uzbekistan, Tashkent, Uzbekistan

**Keywords:** heterozygous familial hypercholesterolemia, CAD, type 2 diabetes mellitus, pcsk9, Uzbek population

## Abstract

**Objective:**

To assess the distribution of PCSK9 E670G genetic polymorphism and PCSK9 levels in patients with Coronary Artery Disease (CAD) and Heterozygous Familial Hypercholesterolemia (HeFH), based on the presence of type 2 Diabetes Mellitus (T2DM).

**Methods:**

The study included 201 patients with chronic CAD, including those with HeFH (n=57, group I) and without it (n=144, group II). DLCN was used to diagnose HeFH. The PCSK9 E670G (rs505151) polymorphism was genetically typed using the PCR-RFLP procedure. In both the patient and control groups, the genotype frequency matched the Hardy-Weinberg equilibrium distribution (P>0.05).

**Results:**

There were twice more G alleles in group I (13, 11.4%) than in group II (17, 6.0%), and thrice more (1, 3.0%) than in the healthy control group; nevertheless, these differences weren’t statistically significant. Simultaneously, PCSK9 levels were higher in HeFH patients (P<0.05) compared to non-HeFH patients not taking statins (n=63). T2DM was equally represented in groups I and II (31.6% *vs.* 33.3%). But carriers of AG+GG genotypes in group I had a higher chance of having a history of T2DM (RR 4.18; 95%CI 2.19-8.0; P<0.001), myocardial infarction (RR 1.79; 95%CI 1.18-2.73; P<0.05), and revascularization (RR 12.6; 95%CI 4.06-38.8; P<0.01), than AA carriers. T2DM was also more common among G allele carriers (RR 1.85; 95% CI 1.11-3.06; P<0.05) in patients with non-HeFH.

**Conclusion:**

T2DM in patients with CAD, both with HeFH and non-HeFH, in the Uzbek population was significantly more often associated with the presence of the “gain-of-function” G allele of the PCSK9 E670G genetic polymorphism.

## 1 Introduction

In our investigation, the distribution of the gain-of-function (GOF) E670G PCSK9 mutation in the patients with HeFH who had prematurely acquired CAD was analysed. As is known, LDL-Cholesterol (LDL-C) has been officially declared the primary cause of Atherosclerotic Cardiovascular diseases (1). PCSK9 GOF mutations significantly increase LDL-C and the risk of CAD through accelerating the degradation of LDL receptors (2). HeFH is a clear example demonstrating the burden of genetically elevated LDL in the world (3).

Another leading cause of CAD is type 2 diabetes mellitus (T2DM): the risk increases by two to four times (4). Up to two-thirds of people with T2DM have Atherosclerotic Cardiovascular Diseases (ASCVD) during their lifetimes (5) and pass away as a result (6). Therefore, the position of leading European guidelines seems well-founded, stating that patients with T2DM should always be considered high-risk or higher (7). Dyslipidemia is the main damaging factor in CAD and T2DM, and an incorrect risk scale leads to frequent underestimation of the disease, resulting in inadequate treatment (8).

As a result, a triangle of issues including CAD, T2DM, and PCSK9 has to be resolved. It is noteworthy that GOF PCSK9 mutations, which initially served as a key discovery in understanding the role of PCSK9 in DLP, including FH (9) subsequently took a backseat to LOF mutations (10), despite their rare occurrence - about 1.5-2.5% in the population (11, 12), and thus their infrequency in HeFH with CAD. However, LOF genetic variants became the prototype for the creation of a generation of targeted PCSK9 therapy drugs, which showed unprecedented efficacy in treating dyslipidemia both in CAD and in CAD with T2DM (13, 14). For the first time, Abifadel et al. (2009) established the modulating effect of the PCSK9 loss-of-function (LOF) genetic variant in FH patients with the LDLR mutation in the Lebanese population, leading to a reduction in LDL cholesterol levels (15). It has also been shown that in FH patients with the LDLR mutation in the Japanese population, the addition of the PCSK9 gain-of-function (GOF) genetic variant is accompanied by an increase in PCSK9 and LDL-C levels (16). Subsequently, a study by Tada et al. (2016) found that the modulatory effects of PCSK9 GOF genetic variants increased the incidence of T2DM (17) compared with patients in whom FH was caused by the LDLR mutation alone (P<0.05).

Warden et al. found a “poor” response to PCSK9 inhibitor therapy in 13.1% of patients, linked to various reasons, including mutations in the PCSK9, LDLR, and other genes (18). Currently, only 1% of patients in Europe receive PCSK9 inhibitors (19). However, the need to expand targeted PCSK9 therapy in the future requires an in-depth study of personalized approaches to their use in both CAD and CAD with diabetes.

The aim of our study was to assess the distribution of PCSK9 E670G genetic polymorphism and PCSK9 levels in patients with CAD and HeFH, based on the presence of T2DM.

## 2 Materials and methods

The study comprised 57 patients with chronic stable CAD and HeFH (I group), who were diagnosed for the first time, and were not taking statins. There were 144 CAD patients in the control group (group II) without HeFH. This group was further divided into subgroup A (n=63), composed of patients who had not taken statins prior to the study, and subgroup B (n=81), made up of patients who regularly took statins at doses prescribed by general practitioners in Uzbekistan: simvastatin 20 mg/day, atorvastatin 10–20 mg/day, rosuvastatin 10 mg/day.

To diagnose HeFH, we used the Dutch Lipid Clinic Network Criteria (DLCN), which is recommended by the European Atherosclerosis Society (EAS) (20). The study included 35 patients with clinically verified probable HeFH scores (6–8). In 21 cases, HeFH was confirmed by an LDL-R gene mutation and in 1 case by an ApoB mutation.

Patients with unstable angina, myocardial infarction in the previous 3 months, chronic renal and hepatic insufficiency, atrial fibrillation, life-threatening ventricular arrhythmias, chronic heart failure above functional class I (NYHA) were not included in the study.

The following clinical and functional parameters were studied in all patients to verify the diagnosis of chronic stable CAD and determine inclusion and exclusion criteria for the study: complaints, family history, medical history, physical examination, presence of T2DM, 12-lead ECG, echocardiography (EchoCG), 24-hour Holter ECG monitoring, exercise stress test.

### 2.1 Biochemical tests

Determination of lipid profile. Blood sampling was carried out on the next day after admission to hospital, in the morning, after 12 hours of fasting, from the cubital vein, in the horizontal position of the patient. Determination of blood lipid levels – total cholesterol (TC), high density lipoprotein (HDL), triglycerides (TG) - performed by enzymatic method on biochemical analyzer “Daytona” (RANDOX, UK). The concentration of LDL-C was determined by the Fridvald’s formula: LDL-C=TC-HDL-TG/5 (mg/dl).

Concentration of high-sensitive C-reactive protein (hsCRP) was determined by a highly sensitive particle-enhanced turbidimetric immunoassay method with latex amplification using “Daytona” (RANDOX, UK).

The level of PCSK9 in the blood was measured using an enzyme-linked immunoassay, utilizing a commercially available “Human Proprotein Convertase 9/PCSK9 ELISA Kit” (MULTI SCIENCE, China).

### 2.2 Determination of genotypic frequencies

The genotypic frequency at the PCSK9 E670G (rs505151) polymorphism was determined using the PCR-RFLP method (21). To amplify the polymorphic gene sequences of E670G of the PCSK9 gene, the following forward primer 5′- CAC GGT TGT GTC CCA AAT GG -3′ and reverse primer 5′- GAG AGG GAC AAG TCG GAA CC -3′ were used.

The PCR reaction was conducted in a 10 μl mixture containing 1X reaction buffer, 1.5mM MgCl2, 0.2mM dNTP, 0.3 μM primers, 25 ng template DNA, and 0.5 U Taq DNA polymerase (Applied Biosystems, Foster City, USA). The amplification was carried out using a GeneAmp 9700 (Applied Biosystems, Foster City, USA), with an initial denaturation at 94°C for 3 minutes, followed by 35 cycles each of 30 seconds at 94°C, 30 seconds at 55°C, and 30 seconds at 72°C, plus a final extension of 5 minutes at 72°C. The specific amplified fragment was 440 bp long. To identify the E670G polymorphism of the PCSK9 gene, the amplified PCR product was subjected to restriction digestion. The reaction was carried out in a volume of 10 μl, containing 5 μl of the PCR product, 1 μl of the 10x restriction buffer, 0.2 U of the restriction endonuclease Bst6I (OOO SibEnzyme, Novosibirsk), and sterile, non-ionized water. Digestion was performed at 65°C for 16 hours.

After digestion, 5 μl of the reaction mixture was run on a 2% agarose gel for 30 minutes at a constant voltage of 100 V in 0.5x TBE buffer. After staining with ethidium bromide, the fragments were visualized using a gel photo-documentation system. Genotyping was performed as follows: a 440 bp fragment corresponded to the GG genotype, fragments of 150 bp and 290 bp represented the AA genotype, and heterozygotes (AG) had fragment sizes of 440, 290, and 150 bp.

### 2.3 Statistical analysis

Statistica 10.0 advanced statistical analysis package was used. The collected data was shown as mean and standard deviation (m ± SD). The statistical importance of the noted measurements for the compared mean values were determined using the Student’s t-test (t) with a calculated error probability (p) to confirm the normality of the distribution. If the distribution of studied variables varied from the normal distribution, then non-parametric analysis tests were made. Specifically, the Wilcoxon signed-rank test was used for comparing two related samples, matched samples, or repeated measurements, and the Mann-Whitney U test was used for comparing the two samples. For searching differences between qualitative statistical measures, the χ2 method was used in simultaneity with Fisher’s exact test for samples that were smaller. The empirical genotype frequency distribution’s conformity to the theoretically expected Hardy-Weinberg equilibrium was estimated using the χ2 test.

## 3 Results

Patients with CAD and HeFH were younger than those in the comparison group (P<0.001), but they often had a history of MI (P<0.05), stroke (P<0.05), and PCI (P<0.05, **Table 1**). T2DM was present in 66 patients and was equally represented between groups I and II (31.6% *vs.* 33.3%).

**Table 1:** Baseline clinical and demographic characteristics in I (HeFH) and II (non-HeFH) groups.

Parameters	I (n=57)	II (n=144)
Age	49.3 ± 9.9***	61.4 ± 9.7
Gender male/female, n (%)	24/33 (42/58)	70/74 (49/51)
AH, n (%)	52 (91.2)	126 (87.5)
T2DM, n (%)	18 (31.6)	48 (33.3)
History of MI, n (%)	30 (52.6)*	42 (29.2)
History of IS, n (%)	6 (10.5)*	4 (2.8)
CABG, n (%)	5 (8.8)	10 (6.9)
PCI, n (%)	18 (31.6)*	24 (16.7)

*,*** - P<0,05, P<0,001 between groups I and II; AH, Arterial Hypertension; MI, Myocardial Infarction; IS, Ischemic stroke; T2DM, Type 2 Diabetes mellitus; CABG, Coronary artery bypass grafting; PCI, Percutaneous coronary intervention.

In group I, among patients with HeFH, Total (P<0.001) and LDL cholesterol (P<0.001) were significantly higher than in group II. Additionally, the PCSK9 level was higher (P<0.05) in I group as opposed to subgroup IIA, which consisted of patients who had not used statins before the study began. Subgroup IIA demonstrated increased levels of Total cholesterol (P<0.01), LDL-C (P<0.01), and hsCRP (P<0.05) when set against subgroup IIB, comprising patients who were on statins before entering the study (**Table 2**).

**Table 2:** Baseline lipid levels and studied biomarkers in I (HeFH) and II (non-HeFH) groups.

Parameters	I (n=57)	II (n=144)		
		All	IIA (n=63), no statins	IIB (n=81), with statins
TC, mg/dL	354.8 ± 80.2***	178.6 ± 42.9	206.3 ± 36.3^^	154.0 ± 29.7
TG, mg/dL	204,5 (151-345)	153.0 (111-239)	165.5 (112-251)	144.0 (108-231)
HDL-C, mg/dL	48.1 ± 12.3	41.6 ± 9.8	41.6 ± 10.8	41.4 ± 9.3
LDL-C, mg/dL	232.5 ± 78.3***	98.4 ± 37.1	124.8 ± 32.4^^	78.1 ± 27.0
Glucose, mmol/l	5.3 (5.0-6.2)	5.4 (5.1-6.2)	5.4 (5.1-5.8)	5.3 (5.1-6.4)
hsCRP, mg/l	6.0 (5-11)	5.3 (2.4-7.3)	5.7(1.9-7.9) ^	5.2 (2.6-7.0)
PCSK9, ng/ml	643.5^#^ (481-797)	592.0 (475-789)	566.0 (439-717)	632.0 (481-822)

***P<0,001, between groups I and II; ^, ^^P<0,05, P<0,01, between subgroups IIA and IIB; ^#^P<0,05, between I and IIA; **TC**, total cholesterol; TG, triglycerides; HDL-C, high-density lipoprotein cholesterol; LDL-C, low-density lipoprotein cholesterol; hsCRP, high-sensitive C-reactive protein.

Both the patient group and the healthy individual’s genotype frequencies for the PCSK9 E670G (rs505151) polymorphism were consistent with the Hardy-Weinberg equilibrium (P>0.05).

The occurrence of G alleles was doubled in group I (13, representing 11.4%) when compared to group II (17, at 6.0%), and tripled in comparison to the healthy Uzbek population (1, or 3.0%). These differences, however, weren’t statistically significant (**Table 3**).

**Table 3:** Distribution of genotypes and alleles of PCSK9 E670G (rs505151) polymorphism in I (HeFH), II (non-HeFH) and III (healthy Uzbek persons) groups, n (%).

Genotypes and alleles	I (n=57)	II (n=144)	All pts (n=201)	III (n=17)
АА	46 (80.7)	128 (89.0)	174 (86.6)	16 (94.1)
AG	9 (15.8)	15 (10.3)	24 (11.9)	1 (5.9)
GG	2 (3.5)	1 (0.7)	3 (1.5)	0
A	101 (88.6)	271 (94.0)	372 (92.5)	33 (97.0)
G	13 (11.4)	17 (6.0)	30 (7.5)	1(3.0)

We looked at the clinical traits of patients based on whether they carried the “damaging” G allele since it is a “gain-of-function” mutation that is uncommon (**Table 4**). Among HeFH patients who were carriers of the AG+GG genotypes, a presence of T2DM (RR 4.18; 95% CI 2.19-8.0; P<0.001), history of myocardial infarction (RR 1.79; 95% CI 1.18-2.73; P<0.05), and any revascularization (RR 12.6; 95% CI 4.06-38.8; P<0.01) were significantly more common. T2DM was also more common among G allele carriers (RR 1.85; 95% CI 1.11-3.06; P<0.05) in patients with non-HeFH.

**Table 4:** Baseline clinical and demographic parameters of patients in I (HeFH) and II (non-HeFH) groups depending on the carriage of the G allele.

Parameters	I (n=57)		II (n=144)	
	AА (n=46)	АG + GG (n=11)	AA (n=128)	АG + GG (n=16)
Age	48.7 ± 10,1	51.8 ± 9,0	61.6 ± 9.8	59.4 ± 9.0
Gender (male/female), n (%)	17/29 (37/63)	7/4 (64/36)	59/69 (46/54)	11/5 (69/31)
AH, n (%)	41 (89)	11 (100)	111 (87)	15 (94)
T2DM, n (%)	9 (19.6)	9 (82.0)	39 (30.5)	9 (56.0)
	RR 4.18; 95% CI 2.19-8.0; P<0.001		RR 1.85; 95% CI 1.11-3.06; P<0.05	
History of MI, n (%)	21 (45.6)	9 (82.0)	37 (29.0)	5 (31.0)
	RR 1.79; 95% CI 1.18-2.73; P<0.05		RR 1.08; 95% CI 0.50-2.35	
History of IS, n (%)	3 (6.5)	3 (27.3)	3 (2.3)	1 (6.2)
	RR 5.4; 95% CI 0.92-31.5		RR 2.67; 95% CI 0.30-24.1	
History of revascularization, n (%)	14 (24.6)	9 (81.8)	29 (22.7)	5 (31.3)
	RR 12.6; 95% CI 4.06-38.8; P<0.01		RR 1.38; 95% CI 0.62-3.05	

RR, relative risk; CI, confidence interval.

For an objective comparison of lipid and PCSK9 levels depending on the distribution of genotypes, we selected subgroup IIA of patients with non-HeFH (n=63) who did not take lipid-lowering therapy before inclusion in the study (**Table 5**). This decision was made because statins can contribute to an increase in PCSK9 levels by lowering lipid levels.

**Table 5:** Baseline levels of lipids and studied biomarkers in I (HeFH) and IIA (non-HeFH, statin free) groups depending on the carriage of the G allele.

Parameters	I (n=57)		IIA (n=63)	
	AА (n=46)	АG+GG (n=11)	AA (n=54)	АG+GG (n=9)
TC, mg/dL	356.1 ± 70.9***	334.4 ± 70.9***	211.2 ± 33.2	192.3 ± 33.5
TG, mg/dL	234,5 (144-359)	191 (158-310)	168 (111-273)	152 (116-159)
HDL-C, mg/dL	48.0 ± 12.9	48.3 ± 10.1	42.9 ± 10.4	41.1 ± 6.8
LDL-C, mg/dL	226.4 ± 80.0***	240.0 ± 58.1***	125.8 ± 29.6	120.7 ± 29.2
Glucose, mmol/l	5.0 (5-6)	7 (5-12)^	5.5 (5-6)	6.8 (5-9)
hsCRP, mg/l	6.8 (5-12)	6.9 (4-9)	6.1 (1-8)	5.0 (2-6)
PCSK9, ng/ml	604 (432-810)	704 # (518-1216)	539 (396-597)	584 (469-763)

***P<0,05, P<0,01, P<0,001 between groups I and IIA; ^P<0,05 between AA and G carriers within groups; #P<0,05 between I(AG+GG) and IIA(AA).

The levels of Total (P<0.001) and LDL cholesterol were, as expected, higher in group I than in group IIA. In group I, the median glucose level was higher in AG+GG carriers, as they were significantly more likely to have T2DM.

The variability of the PCSK9 level and the relatively small number of carriers of the AG+GG genotype in groups I and II made it challenging to identify significant differences in PCSK9 concentration within the groups. However, the distribution median was nominally higher in the carriers of the G allele. Importantly, the level of PCSK9 was significantly higher in carriers of the AG+GG genotype in group I compared to AA carriers in subgroup IIA, although AA carriers in group I did not demonstrate such an effect.

## 4 Discussion

The results of our study showed an assoсiation between the carriage of “gain-of-function” G allele of the PCSK9 E670G genetic polymorphism and the incidence of T2DM in CAD patients with HeFH and non-HeFH, in the Uzbek population. Obviously, this assoсiation may indicate the influence of genetically elevated PCSK9 on the development of coronary artery disease and T2DM simultaneously.

In the Dallas Heart Study (22), which was a large multi-ethnic study, PCSK9 levels varied over a substantial 100-fold range (33–2988 ng/ml; median, 487 ng/ml). This variation depended on factors such as gender, metabolic status, and genetics. Specifically, PCSK9 levels were found to be higher in individuals with metabolic syndrome, insulin resistance, and elevated fasting glucose levels. A wealth of subsequent studies confirmed that an increase in circulating PCSK9 is associated not only with hypercholesterolemia but also with insulin resistance, increased glucose levels, and diabetes mellitus (23–25).

However, Saavedra et al. (26) genotyped 724 patients with FH and found “loss-of-function” (LOF) PCSK9-InsLEU variant in 26%. At that, pre-diabetes and diabetes mellitus were more often observed in InsLEU-carrier group compared with non-carriers (10% *vs.* 6%, P=0.04). InsLEU carriage was not accompanied by a decrease in lipids, however, FH patients with LOF mutation had fewer coronary events (22.1% *vs.* 30.6%, P=0.04). The assessment of pre-diabetes and diabetes was extracted from an electronic patient database, where the diagnoses were formulated by the responsible physicians, following the ADA’s protocols (American Diabetes Association). Impaired fasting glucose (IFG) was evaluated according to the same guidelines, with a glucose cut-off value of ≥5.6 mmol/l.

Lorenzo Da Dalt et al. (27) have shown in the experimental study that genetic deletion of PCSK9 in mice contributes to impaired glucose tolerance due to impaired insulin secretion, but not insulin resistance. This could possibly be explained by an increase in LDL receptor activity in pancreatic beta-cells in response to decreased PCSK9, with subsequent cholesterol accumulation, cellular dysfunction, and impaired insulin secretion.

In contrast, a group of French researchers by Langhi et al. (28) showed that PCSK9 is localized in human pancreatic d-cells, is not expressed in beta-cells, and is also not secreted by isolated murine islets in culture. Fasting glucose levels were similar in their experiment between PCSK9+/+ and PCSK9_/_ mice. Also, in some other studies, LOF PCSK9 variants InsLEU (1745 healthy French-Canadian individuals) and R46L (5972 French individuals) did not demonstrate an effect on glucose homeostasis and insulin resistance (29, 30).

Randomized controlled trials investigating the safety and efficacy of evolocumab (31) and alirocumab (32) in patients with and without T2DM also do not indicate an increase in glucose level, glycated hemoglobin and an increased incidence of T2DM compared to standard therapy, however, it may be necessary to wait for long-term results of studies.

In 2005, Chen et al. (31) studied GOF genetic polymorphisms PCSK9 in patients who participated in LCAS study of fluvastatin efficacy (n=372) and in another independent population in TexGen study (n=319). They found that carriage of G-allele of the E670G was associated with an increase in LDL-C and coronary atherosclerosis. In the LCAS study, which predominantly involved Caucasians (90%), the G allele frequency stood at 7.4%. Meanwhile, in the TexGen study with 79% Caucasians, the G allele frequency was 4.4%.

Another study by Evans and Beil (32), which encompassed 506 patients attending the University of Hamburg’s Lipid Clinic, found G allele carriers at an average frequency of 5%. However, in individuals with LDL-C levels above the 95th percentile, which consisted of patients with FH and polygenic hypercholesterolemia, the G allele frequency rose to 6.4%. CAD in G-carriers was significantly more common among men, but not among women.

In our research involving an Uzbek population of 144 CAD patients, the G allele frequency was 6.0% (17 pts) but, this rate was doubled to 11.4% (13, including 2 GG) among the 57 FH patients. Data from large meta-analyses conducted in recent years confirm the association of carriage of the E670G G allele with increased lipid levels and the risk of CAD and MACE (**Table 6**).

**Table 6:** Effect of E670G PCSK9 Polymorphism on Lipids, ASCVD and MACE from Large Meta-Analyses.

Authors	Number of studies	Effect on lipids	Effect on ASCVD	Ref.
Cai et al. (2015)	Meta-analysis based on 17 studies	Сholesterol levels among carriers of the G-allele is higher than non-carriers (P=0.004)	Risk of CAD and MACE among carriers of the G-allele is higher than in non-carriers (OR 1.601, 95% CI 1.314-1.951, P<0.001).	(33)
Qiu et al. (2017)	Meta-analysis of 15 studies comprising 14,451 cases	Аssociation between G-allele carriage and increases in triglycerides and LDL-C	High cardiovascular risk (OR 1.50, 95% CI 1.19-1.89, P=0.0006).	(34)
Li et al. (2020)	Meta-Analysis of 5,484 cases from 13 studies	TC and LDL-C levels in the GG genotype group were much higher (P < 0.05)	Association between PCSK9 gene E670G polymorphism and CAD (OR1.79, 95% CI 1.42–2.27, P = 1.00 × 10^-6^)	(35)

The association of E670G PCSK9 Polymorphism with T2DM has been established in several studies in patients of Asian ethnicity including with FH in recent years (**Table 7**).

**Table 7:** Studies showing an association between the E670G polymorphism of PCSK9 and T2DM.

Authors	Характеристика обследованных	Effect on lipids	Effect on ASCVD	Effect on T2DM	Ref.
Slimani et al. (2014)	258 Tunisian patients, CAD in 192 pts (stenosis ≥50% in at least one coronary artery) and 66 without CAD. 232 healthy individuals in the comparison group (without obesity, hypertension, dyslipidemia)	LDL-C in AG+GG CAD pts more (P=0.001)	Association E670G polymorphism with the risk of CAD, DM and Ishemic stroke (IS).	Carriage of G allele significantly higher in T2DM than in non-DM subgroup (0.151 *vs*. 0.091, p=0.036).	(36)
Tada et al. (2016)	42 FH Japanese patients with a *PCSK9 GOF* mutation *vs.* 198 FH patients with a *LDLR* mutation	LDL-C is higher in FH-PCSK9 *vs.* FH-LDLR (p<0.001)	The incidence of CAD did not differ in both FH groups	T2DM more common in FH-PCSK9 than FH-LDLR (p<0.001)	(17)
Chiang et al. (2020)	501 Taiwanese pts with CAD *vs.* 334 controls without CAD	not detailed	CAD was significantly higher in pts carrying the AG+GG	Frequency of T2DM significantly higher in pts with AG+GG genotype (73.3%) *vs.* AA (39.2%)	(37)
Mohamed et al. (2021)	112 Egyptian individuals without weight gain compared to 100 T2DM patients without CAD and 84 T2DM patients with CAD	AG genotype among Egyptian T2DM pts Positively associated with atherogenic index of plasma.	AG genotype are cardiovascular disease risk factors among Egyptian T2DM pts	The prevalence of the E670G AG in diabetics with CAD was significantly greater than the other two groups	(38)

In patients with CAD, in the Nothern Afrika and Asian populations (Tunisia, Egypt, Taiwan, Uzbekistan) in addition to elevated LDL-C and TG, the carriage of PCSK9 G-allele is associated with T2DM in some studies. This is consistently associated with increases in LDL cholesterol, triglycerides (TG), and CAD. Recent research confirms that an increase in circulating PCSK9 levels not only accompanies hypercholesterolemia but also leads to higher glucose levels, insulin resistance, and T2DM.

It can be observed that, unlike in the European population, the carriage of the G-allele E670G (rs505151) of PCSK9 genetic polymorphism in the Asian population occurs 2-3 times more frequently. Krittanawong et al. (2022) suggest that to optimize doses of PCSK9 targeted therapy, the PCSK9 R46L alleles should be tested in Europeans, while the PCSK9 E670G polymorphism should be considered in Asian populations (39). Considering recent research, T2DM should be considered another essential player in the selection of personalized therapy.

### 4.1 Conclusion

T2DM in patients with CAD, both with HeFH and non-HeFH, in the Uzbek population was significantly more often associated with the presence of the “gain-of-function” G allele of the PCSK9 E670G genetic polymorphism. This may have implications for the development of personalized PCSK9 targeted therapy in patients with CAD and T2DM.

### 4.2 Study limitation

The present study does have some limitations. It includes a relatively small number of patients with CAD (n=201), comprised of 57 patients with FH and 144 without FH. Among these, 30 G alleles were identified in 27 carriers (13.4%). Further risk stratification for this genetic marker is warranted, particularly among patients with coronary artery disease, diabetes mellitus, and FH.

## Data availability statement

The original contributions presented in the study are included in the article/supplementary material. Further inquiries can be directed to the corresponding author.

## Ethics statement

The studies involving humans were approved by Ethical Committee of the Republican Scientific and Practical Medical Center for Cardiology of the Ministry of Health of the Republic of Uzbekistan. The studies were conducted in accordance with the local legislation and institutional requirements. The participants provided their written informed consent to participate in this study.

## Author contributions

RA: Writing – review & editing, Conceptualization, Data curation, Funding acquisition, Project administration, Visualization, Writing – original draft. AS: Writing – original draft, Conceptualization, Validation. AA: Writing – review & editing, Methodology. KF: Writing – review & editing, Supervision. SK: Writing – original draft, Project administration, Validation. GA: Investigation, Writing – original draft. DZ: Writing – original draft, Methodology. RK: Writing – original draft, Funding acquisition, Project administration. LK: Data curation, Writing – original draft, Investigation. AK: Software, Writing – original draft, Data curation, Investigation.
